# Smartphone-based turbidity reader

**DOI:** 10.1038/s41598-019-56474-z

**Published:** 2019-12-27

**Authors:** Hatice Ceylan Koydemir, Simran Rajpal, Esin Gumustekin, Doruk Karinca, Kyle Liang, Zoltan Göröcs, Derek Tseng, Aydogan Ozcan

**Affiliations:** 1 Biengineering Department, University of California Los Angeles, Los Angeles, 90095 CA USA; 2 Electrical and Computer Engineering Department, University of California Los Angeles, Los Angeles, 90095 CA USA; 3 California NanoSystems Institute, Los Angeles, 90095 CA USA; 4 Microbiology, Immunology and Molecular Genetics, University of California Los Angeles, Los Angeles, 90095 CA USA; 5 Computer Science Department, University of California Los Angeles, Los Angeles, 90095 CA USA

**Keywords:** Biomedical engineering, Electrical and electronic engineering, Imaging and sensing

## Abstract

Water quality is undergoing significant deterioration due to bacteria, pollutants and other harmful particles, damaging aquatic life and lowering the quality of drinking water. It is, therefore, important to be able to rapidly and accurately measure water quality in a cost-effective manner using e.g., a turbidimeter. Turbidimeters typically use different illumination angles to measure the scattering and transmittance of light through a sample and translate these readings into a measurement based on the standard nephelometric turbidity unit (NTU). Traditional turbidimeters have high sensitivity and specificity, but they are not field-portable and require electricity to operate in field settings. Here we present a field-portable and cost effective turbidimeter that is based on a smartphone. This mobile turbidimeter contains an opto-mechanical attachment coupled to the rear camera of the smartphone, which contains two white light-emitting-diodes to illuminate the water sample, optical fibers to transmit the light collected from the sample to the camera, an external lens for image formation, and diffusers for uniform illumination of the sample. Including the smartphone, this cost-effective device weighs only ~350 g. In our mobile turbidimeter design, we combined two illumination approaches: transmittance, in which the optical fibers were placed directly below the sample cuvette at 180° with respect to the light source, and nephelometry in which the optical fibers were placed on the sides of the sample cuvette at a 90^°^ angle with respect to the to the light source. Images of the end facets of these fiber optic cables were captured using the smart phone and processed using a custom written image processing algorithm to automatically quantify the turbidity of each sample. Using transmittance and nephelometric readings, our mobile turbidimeter achieved accurate measurements over a large dynamic range, from 0.3 NTU to 2000 NTU. The accurate performance of our smartphone-based turbidimeter was also confirmed with various water samples collected in Los Angeles (USA), bacteria spiked water samples, as well as diesel fuel contaminated water samples. Having a detection limit of ~0.3 NTU, this cost-effective smartphone-based turbidimeter can be a useful analytical tool for screening of water quality in resource limited settings.

## Introduction

Algae, bacteria, pollutants, and other harmful particles deteriorate water quality^1,2^. These contaminants significantly affect surface water and drinking water, and may even cause aquatic life degradation^3,4^. Partially related to contamination and lack of proper infrastructure, there is limited access to clean water in underdeveloped countries. For example, in several African countries, less than 50% of the population has access to clean water^5^. Thus, it is imperative for individuals in these countries to assess and monitor water quality in a rapid and cost-effective way. Despite this urgent need, most of the currently existing technologies that measure water quality require the samples to be collected and transported back to a central lab. There are also field-portable devices that can measure water quality at the stream bed^6–8^, however, these devices cost hundreds of dollars and are not easily accessible in underdeveloped countries.

Turbidity is a vital parameter used to measure water quality. It is a broad measure of particles in a solution. According to the Environmental Protection Agency (EPA), less than 1 NTU is the maximum allowable turbidity that water has if there are conventional filtration systems^9^. Water turbidity can be up to 5 NTU if there are additional types of filtration systems such as slow sand and diatomaceous earth filtration^9^. Furthermore, aquatic life can only survive in streams if the turbidity is under 25 NTU and if it is less than 50 NTU in lakes and reservoirs^9^. Governments also set standards for the turbidity of wastewater that water treatment facilities are allowed to dump into a sea or an ocean. For example, there is a limit of 75 NTU for the monthly average of the effluent water and a 100 NTU limit for the weekly average of the effluent water in California (USA)^10^. These standards are important to ensure that we have appropriately clean drinking water and that the aquatic life continues to flourish. In order to maintain these standards at a global scale, reliable and mobile methods that can rapidly and accurately measure turbidity are needed.

**Figure 1:**
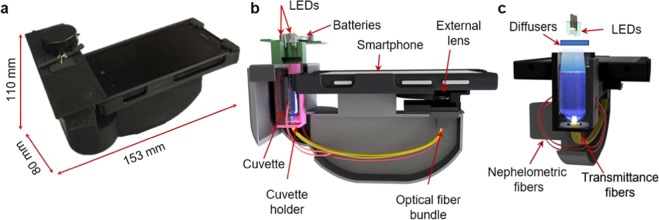
Smartphone based turbidimeter. (**a**) A photograph of the device. (**b**) Schematic of the device. (**c**) Schematic of the illumination path and the locations of the optical fibers for two illumination modes (i.e., nephelometry and transmittance).

**Figure 2:**
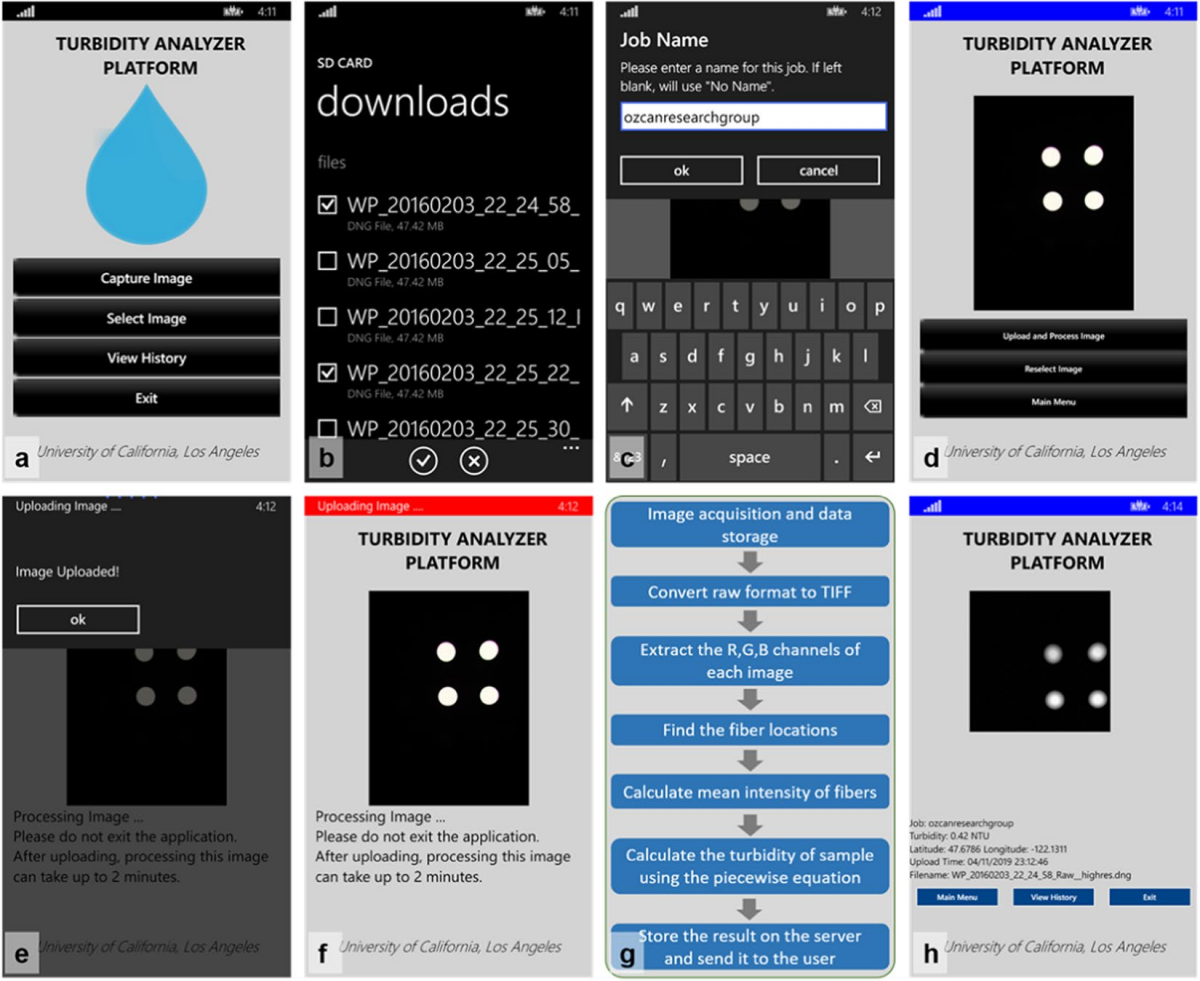
Turbidity measurement steps using a custom developed smartphone application. (**a**) Main menu of the application. It shows four buttons that allow the user to capture an image, select an image, view history or exit the application (**b)** A screenshot of the application after two raw format images from the photo library are selected. (**c**) After assigning a job name to a measurement (**d**) the selected images are ready to be uploaded and processed at our servers. (**e**) The warning box that displays the screen after the images are uploaded to the servers. (**f**) The application informs the user about the status of the images at the server. (**g**) Image processing steps performed at the server. (**h**) The result of a turbidity measurement. It displays the turbidity level in NTU, along with the GPS location of where the image was captured at, the upload time and the image name.

Standard turbidimeters quantify water turbidity by measuring the scattering and absorption of light due to foreign particles suspended in a solution. Most EPA-certified turbidimeters operate by making use of nephelometry and transmittance. Here, we present a field-portable and cost-effective smartphone-based reader to quantify the turbidity of water samples in resource limited settings. This optical reader utilizes inexpensive optical parts, including an external lens for image formation, optical fibers and two white light emitting diodes (LEDs), enclosed in a 3D-printed attachment to the camera unit of a smartphone (Fig. 1). The turbidity of a water sample in a disposable cuvette is quantified by using nephelometry and transmittance readings, obtained through fiber optic cables (Fig. 1b,c). A custom-developed smartphone application is used to transmit the captured images of these fibers to local or remote servers for rapid quantification of the turbidity of the sample (Fig. 2). The digital analysis of turbidity is based on a calibration curve that relates the intensity of the fibers to NTU readings, and in less than 90 s, the result of this analysis is sent back to the user through the same smart application. Compared to earlier work that successfully combined nephelometry with smartphones^11^, our mobile turbidimeter combines nephelometry and transmittance to be able to measure a much wider range of turbidity values, from 0.3 NTU to 2000 NTU, allowing for a more accurate categorization of turbidity levels and water quality for various types of samples.

## Results and Discussion

Our field-portable, smartphone-based turbidimeter weighs ~350 g (including the smartphone) and water turbidity is measured using a 4 mL sample in a glass cuvette that is disposable. Our prototype is cost-effective, with its parts costing only ~$45 (excluding the smartphone), which can be decreased to less than $10, with large volume manufacturing. While designing the turbidimeter, we considered two major design criteria, defined by EPA Method 180.1: (1) The distance traversed by the incident light and scattered light within the sample cuvette is less than 2 cm in our design, which needed to be less than 10 cm; (2) Our detector, which is the rear camera of the smartphone, receives, as desired, the transmitted light as well as the 90°-scattered light through the fibers (see Fig. S1). The light coming out from two LEDs is scattered using a paper-based diffuser to provide uniform illumination on the sample. The scattered light from the particles in the liquid sample is collected by 4 nephelometric fibers located on the sides of the inner surface of the sample holder (Fig. S1a). The scattered light is then transmitted through these optical fibers, which are imaged by a CMOS image sensor, i.e. the detector in the design (refer to the Methods section for details). Furthermore, the light transmitted through the sample is also collected by 4 separate transmittance fibers located at the bottom of the cuvette (Fig. S1b). Similar to the nephelometric fibers, the light transmitted through these transmittance fibers is imaged by a CMOS image sensor. Based on the acquired images, the turbidity of the sample is calculated using a custom developed smartphone application.

**Figure 3:**
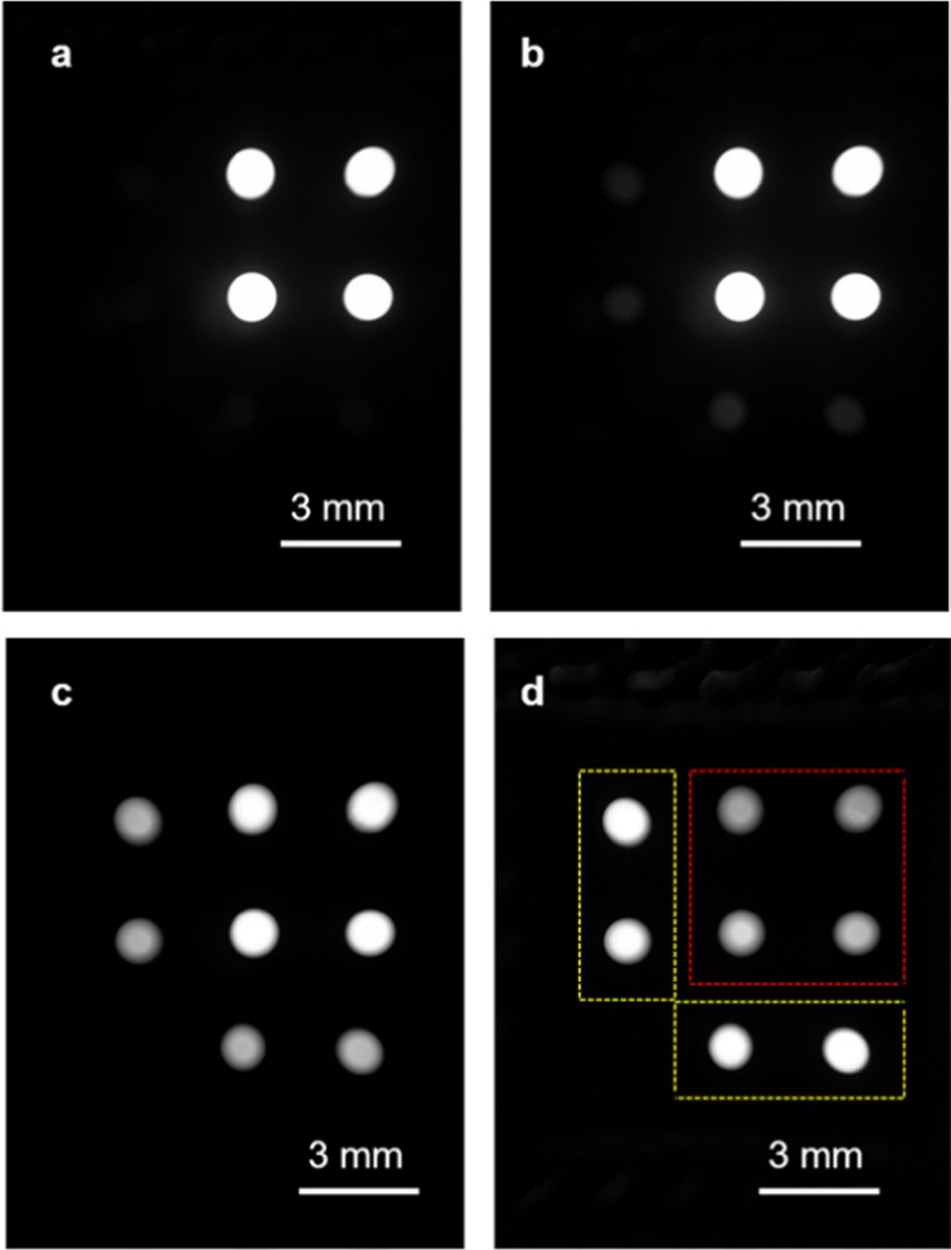
Images of the end facets of the fiber optic cables captured using the portable turbidimeter at different water turbidity levels: (**a**) 0.05 NTU, (**b**) 15 NTU, (**c**) 320 NTU, and (**d**) 750 NTU at an exposure time of 0.8 s. Yellow boxes show the nephelometric fibers and red box shows the transmittance fibers.

We first tested the detection limits of this platform with samples of known turbidities (refer to the Methods section for details). Each sample was imaged using the portable reader at two different exposure times (0.02 s and 0.8 s) and the raw format images were used for analysis. The average signal levels corresponding to the transmittance and nephelometric fibers (Fig. 1c) were calculated automatically using a custom-developed image processing algorithm. Figure 3 shows some sample images captured using the smartphone turbidimeter at two different exposure times for different levels of turbidity. We used the image with the low exposure time (i.e. 0.02 s) to determine the intensity of the transmittance fibers and the image with high exposure time (i.e. 0.8 s) to determine the nephelometric intensity. The lowest turbidity sample that we tested was reagent grade water with a turbidity of 0.05 NTU, for which the intensities at the nephelometric fibers were close to 0 (Fig. 3a). As the turbidity of the sample increased, the nephelometric fibers’ signal became apparent to the naked eye (Fig. 3b–d). Since there was more scattering in the samples at high turbidity levels, the transmitted light through the sample decreased and therefore, the intensities at the transmittance fibers also decreased (Fig. 3c,d).

**Figure 4:**
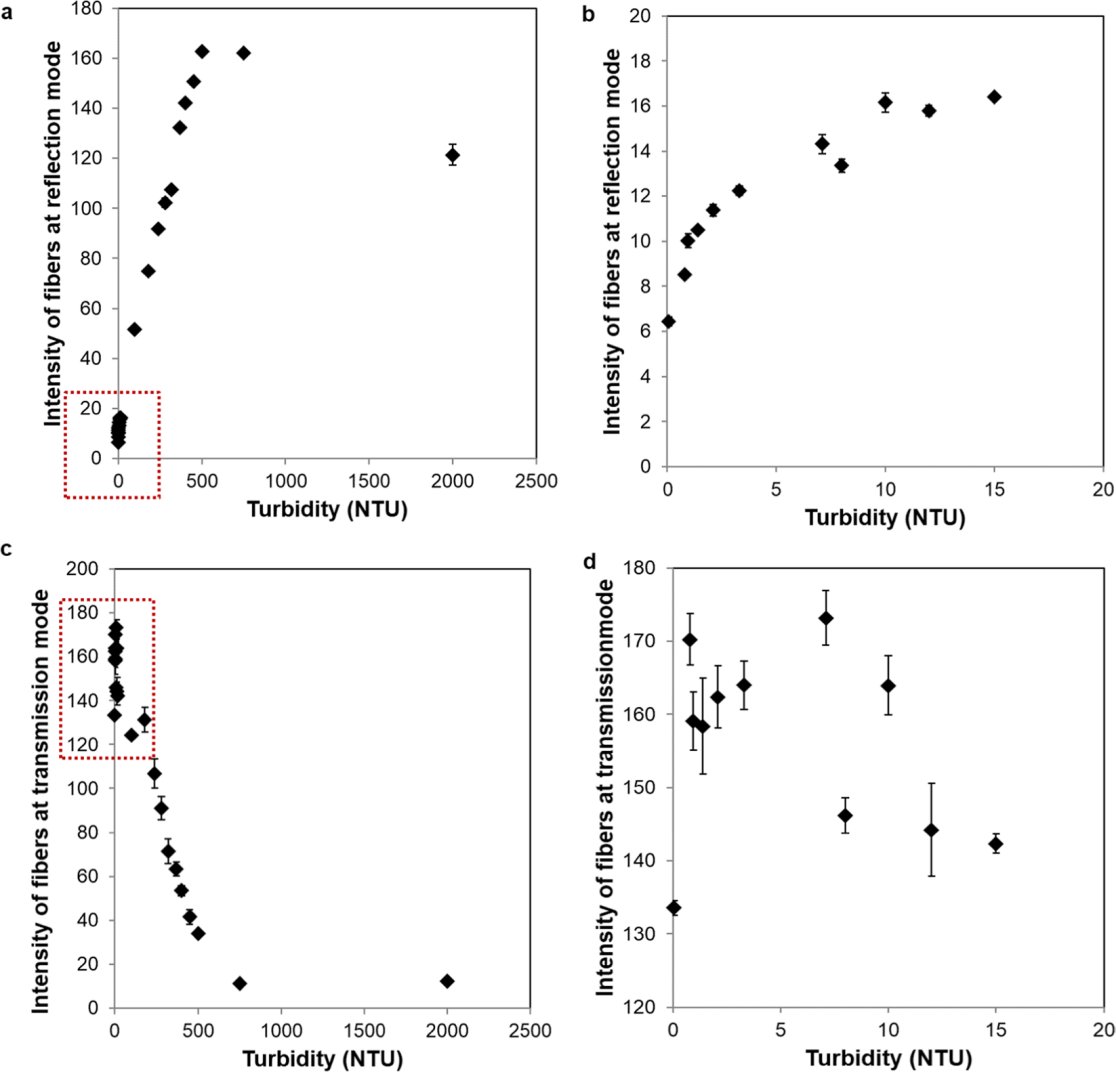
Measured intensities of turbid water samples using (**a**) nephelometry and (**c**) transmittance modes of our mobile turbidimeter. (**b**) Zoomed in version of the red box area in (**a**). (**d**) Zoomed in version of the red box area in (**c**).

**Figure 5:**
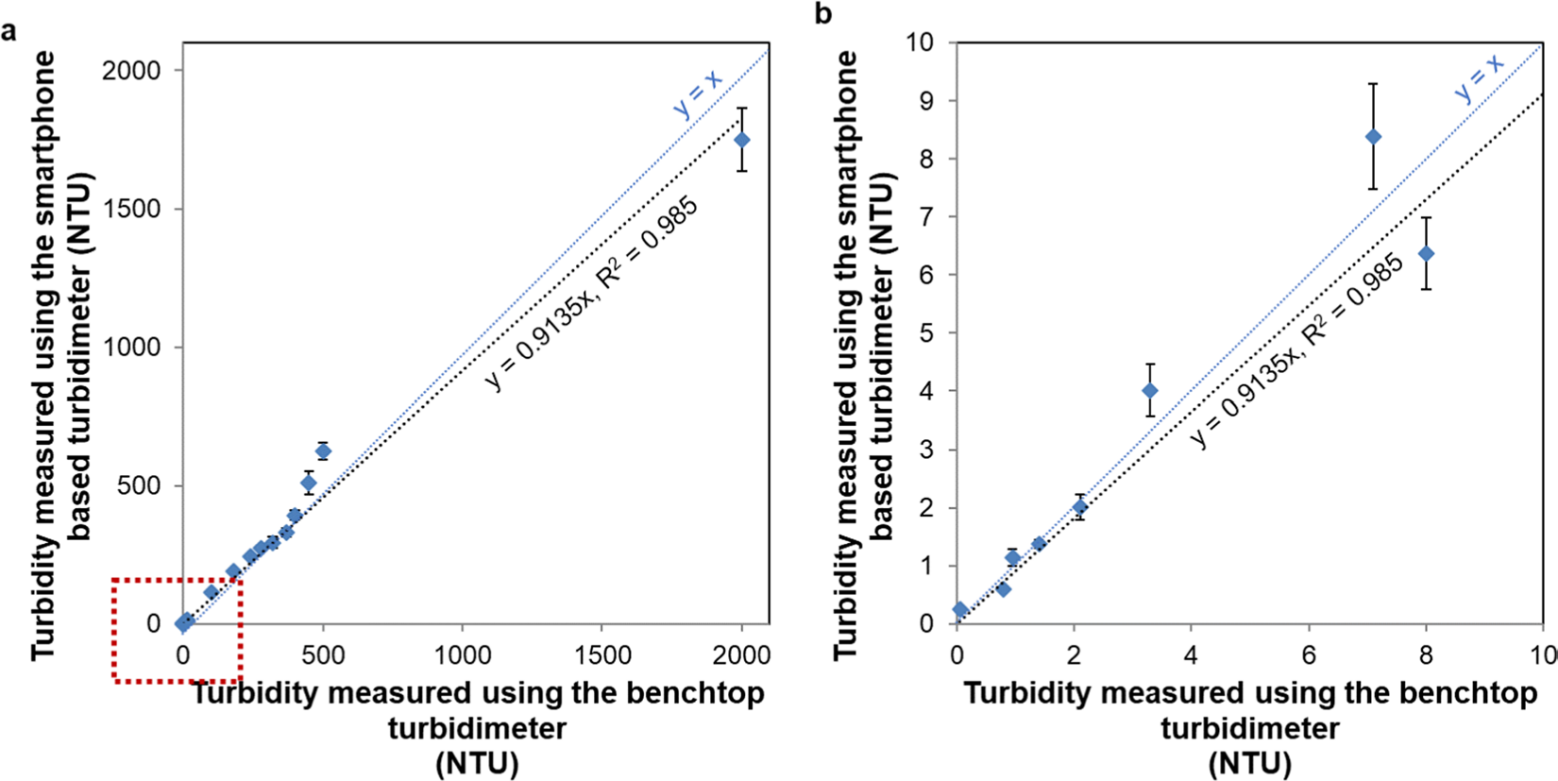
(**a**) Turbidity measurements using the smartphone-based turbidimeter compared against the measurements of an EPA-certified benchtop turbidimeter. (**b**) Zoomed in version of the red box area in (**a**).

Figure 4 demonstrates the relationship between the corresponding fiber intensities and the sample turbidity for each illumination mode. As seen in Fig. 4a,b,d low turbidity samples (<320 NTU) can be more accurately measured with nephelometry since they exhibit a much lower standard deviation. However, beyond 320 NTU, this mode of measurement becomes difficult to use for quantification of turbidity. In fact, such higher turbidity samples can be more accurately measured using the transmittance mode (Fig. 4c), covering a range of 320 NTU to 2000 NTU. Based on the best fit to the experimental results reported in Fig. 4, Eq. (1) describes our calibration curve that converts the average intensity of the fibers to the sample turbidity:

1 $${\rm{Turbidity}}=\{\begin{array}{c}\begin{array}{c}\begin{array}{cc}0.01563\ast \exp (0.4261\ast {I}_{R}) & \,{I}_{R} < 12\,\end{array}\\ \begin{array}{cc}2.1113\ast {I}_{R}-21.858\, & 12\le {I}_{R} < 17\\ 3.1661\ast {I}_{R}-48.853 & 17\le {I}_{R} < 105\end{array}\\ \begin{array}{cc}22400\ast {{I}_{T}}^{-1.016} & {I}_{R}\,\ge 105\end{array}\end{array}\end{array}$$

where ${I}_{R}$ is the average intensity of the reflection mode fibers, ${I}_{T}$ is the average intensity of the transmittance mode fibers, and turbidity is reported in NTU. When we blindly measured the turbidities of unknown samples quantified with the smartphone-based reader by using Eq. (1) and compared our results against the turbidities of the same samples measured using an EPA-compliant benchtop turbidimeter, our platform demonstrated an overall accuracy of 91.35% (see Fig. 5 and Table S2). As can be seen in Fig. 5a, a larger deviation in our measurement results occurs at high turbidities (i.e., ≥500 NTU); we can lower our measurement error for such higher turbidity samples by diluting the water sample of interest before a measurement. A more accurate turbidity of the sample can be then calculated by taking into account the dilution factor^12^ along with our mobile turbidimeter measurement.

The limit of detection of our smartphone-based turbidimeter can be estimated as 0.31 NTU based on the mean turbidity for the control samples (i.e., reagent grade water with a turbidity of 0.05 NTU) plus 3 times the standard deviation (see Table S1)^13^. This limit of detection is also in line with EPA’s requirement for drinking water regulations: for example, according to the Long Term 1 Enhanced Surface Water Treatment Rule (LT1ESWTR), the turbidity measured at the combined filtered effluent and individual filtered effluent of any public water system using surface water or ground water under the direct influence of surface water cannot be higher than 1 NTU at any time and at least 95% of the tested samples in any month cannot be higher than 0.3 NTU^9^. Additionally, the turbidity of drinking water cannot exceed 5 NTU. Therefore, using our smartphone-based turbidimeter, the turbidity of various water samples can also be accurately monitored in line with the turbidity requirements of EPA.

Our smartphone turbidimeter is also useful for monitoring high turbidity levels for various other applications. For example, an increase in water turbidity can be dangerous for fish since it makes it difficult for them to find food and avoid predators. When the turbidity level is increased from its natural state to e.g., 100 NTU or larger, the diversity of plant species decreases, fish start to show signs of stress, and fish egg survival rates decrease^14^. As turbidity increases to e.g., 1000 NTU, the respiration rate of fish increases and their feeding capability decreases^4^. Beyond several thousand NTU, the growth rate of fish is reduced, and they eventually die. Another application, where cost-effective and rapid monitoring of water turbidity is very important, involves water treatment plants. Although raw water turbidity is usually below 10 NTU in normal weather conditions, it can increase to become hundreds of NTU due to e.g., storms and floods. These fluctuations in turbidity levels of raw water determine the design and operation of treatment plants so that they can continue cleaning the water and provide water to the environment at the same quality based on EPA regulations^15^. The large dynamic range of our mobile phone turbidimeter, along with its cost-effectiveness and field-portability, provides us a plethora of applications in which it can be of essential use.

**Figure 6:**
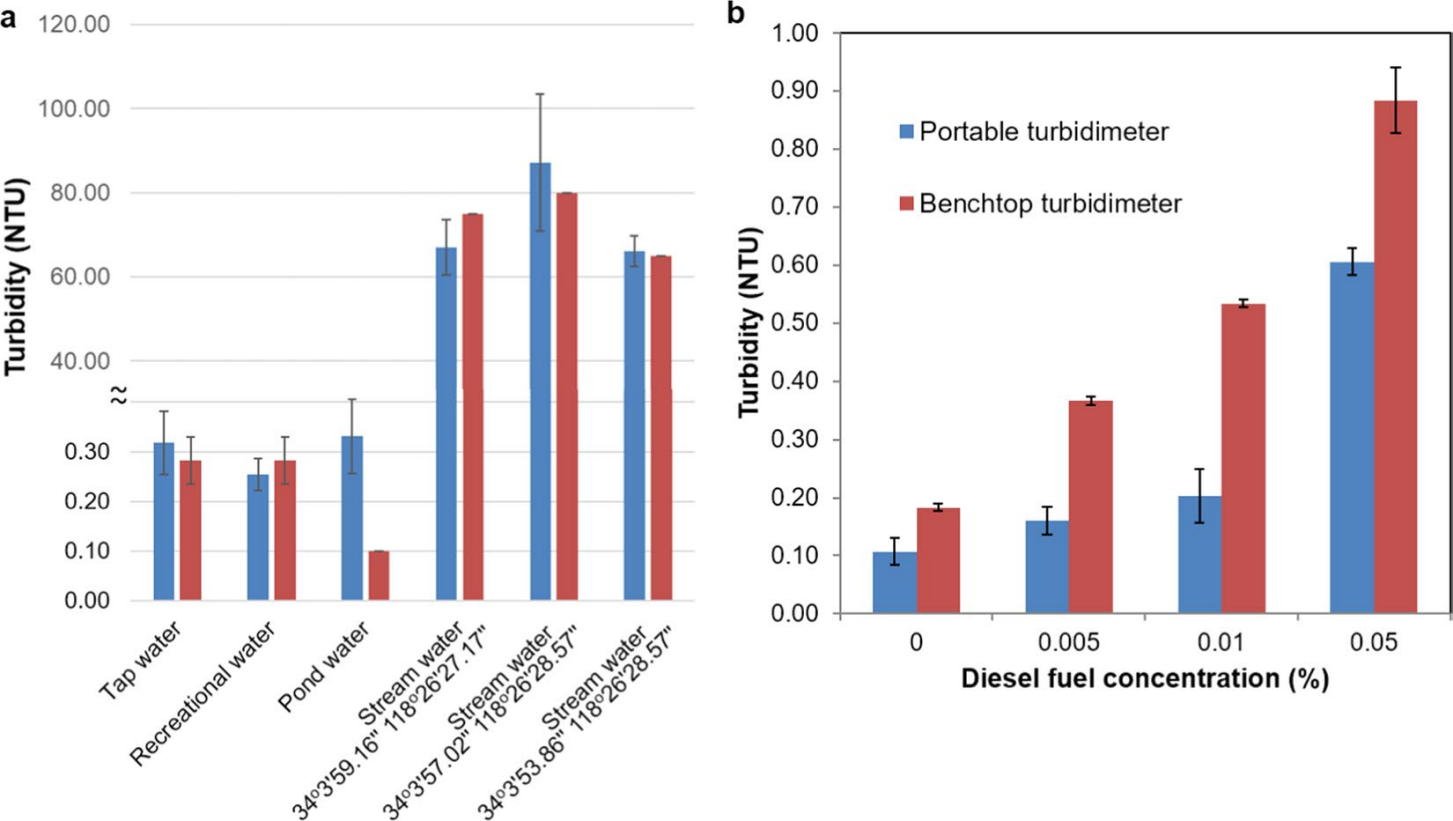
Field testing. (**a**) Turbidity of water samples taken from different sources. (**b**) Turbidity of non-potable water samples spiked with diesel fuel at different concentrations. At both images, blue bar demonstrates the turbidity measured using the smartphone turbidimeter and red bar demonstrated the turbidity measured using the benchtop turbidimeter.

We further tested the performance of our platform using water samples collected from different sources in Los Angeles (USA) (Fig. 6a) including e.g., tap water, recreational water, pond water, and surface water. Our device quantified the turbidity levels of tap water and recreational water to be about 0.3 NTU in accordance with the measurements of an EPA-compliant benchtop turbidimeter. The turbidity of the pond water sample was about 0.1 NTU, which is below the limit of detection of our platform; therefore, the turbidity of this pond water sample was measured as 0.3 NTU using our mobile turbidimeter. Surface water samples at UCLA Mildred A. Mathies Botanical Garden had turbidity levels in the range of 60 NTU to 80 NTU measured using the benchtop turbidimeter, and the turbidity readings for these samples obtained using our mobile turbidimeter provided a good measurement agreement (Fig. 6a).

Another important application that our mobile turbidimeter provides a cost-effective and rapid measurement solution is the detection of fuel contamination in drinking water, which has various adverse effects on the central nervous system^16,17^. Blind testing of our smartphone-based turbidimeter was performed using diesel fuel contaminated water samples as shown in Fig. 6b (refer to Materials and Methods section for details). Although the results obtained from the portable reader are slightly off (~0.3 NTU) compared to those of the benchtop turbidimeter, the trend of the measured turbidity levels as the fuel concentration increases is similar for both turbidimeters.

**Figure 7:**
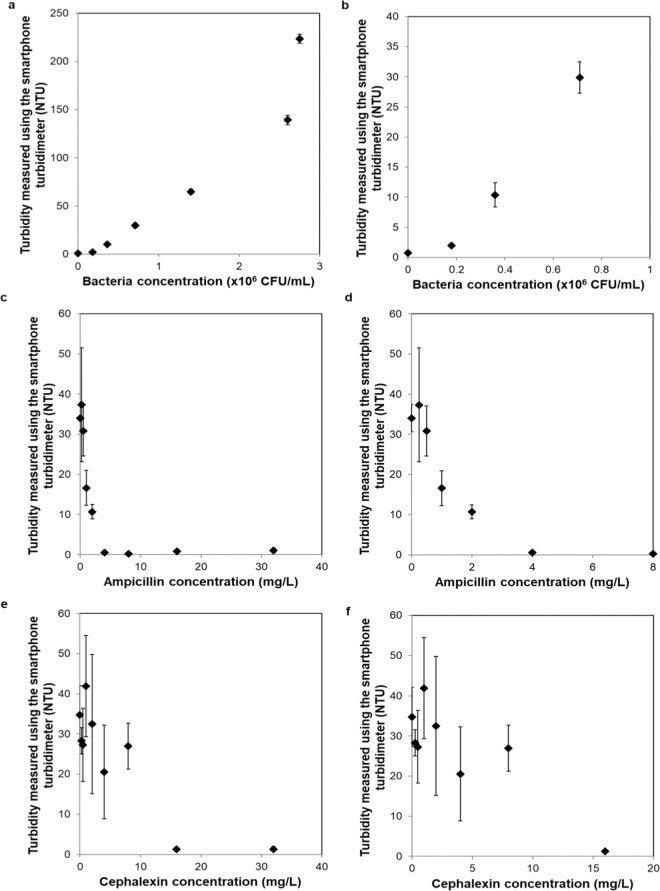
(**a**) Turbidity measured using the smartphone turbidimeter as a function of *E. coli* concentration. (**b**) Zoomed in version of (**a**) for low concentrations of bacteria. (**c**) Turbidity measured using the smartphone turbidimeter as a function of ampicillin concentration. (**d**) Zoomed in version of (**c**) for 0–8 mg/L of ampicillin concentration. (**e**) Turbidity measured using the smartphone turbidimeter as a function of cephalexin concentration. (**f**) Zoomed in version of (**e**) for 0–20 mg/L of cephalexin.

While turbidity is a measure of the suspended particles in water samples and a parameter used to monitor water quality, it can also be an indicator of bacterial growth in a liquid sample. We also tested the performance of our mobile device to monitor bacterial growth using *E. coli* spiked solutions at different concentrations and measured the resulting turbidities. As seen in Fig. 7a,b, with increasing concentrations of spiked bacteria, the corresponding turbidity also increased, as expected. For example, the turbidity of the blank/control sample (phosphate buffer solution) was 0.73 NTU while the turbidity of the contaminated sample containing 0.18 × 10^6^ colony forming unit (CFU)/mL was 1.96 NTU (measured immediately after the spiking of the sample with bacteria). As the concentration of *E. coli* increased to ~3 × 10^6^ CFU/mL, the corresponding turbidity increased to 225 NTU. Therefore, having a limit of detection of 0.3 NTU, our mobile turbidimeter can be used to detect early growth stages of bacteria in liquid media. For example, *E. coli* has a generation time of ~20 min^18^, so, it will take approximately 15 multiplication times (~5 hours) for a single bacterium to reach 0.32 × 10^6^ CFU per mL, which corresponds to ~10 NTU, based on Fig. 7b. If we assume the time interval between the inoculation and the start of the division of the bacterium is 2 h, then the total detection time will be ~7 h.

We also demonstrated the ability of our platform to determine the minimum inhibitory concentration (MIC) of antibiotics using bacterial samples. We used two antibiotics, i.e. ampicillin and cephalexin, to determine the susceptibility of *E. coli* (refer to Materials and Methods section for details). As the antibiotic concentration increased, the turbidity of the sample decreased, as expected (Figs. 7c–f). As shown in Figs. 7d,f, the data point at which the turbidity reached close to zero reports the corresponding MIC value. Our experiments, measured using the smartphone-based turbidimeter, revealed an MIC of 4 mg/L and 16 mg/L for ampicillin and cephalexin, respectively. These values are in good agreement with earlier MIC findings for *E. coli*^19–21^. Our portable turbidimeter has a single cuvette holder, so we needed to measure each sample individually. However, our measurement configuration can also be converted to image and measure the turbidity levels of multiple cuvettes in parallel^22^.

We also compared our portable turbidimeter and its performance against various portable turbidimeters developed by other researchers; see Table 1. Our turbidimeter’s working principle is based on imaging, while various other existing solutions are based on either proximity sensing^11^ or light-to-frequency sensing^23^ which partially increase the cost and the number of required steps to obtain the measurement result. As for the illumination source, we used two white LEDs powered using coin-size batteries, which could also be powered up using the smartphone’s battery. The presented approach can also be used with other smartphone designs after some minor modifications. As detailed in Table 1, our smartphone based turbidimeter has a large dynamic range (0.3–2000 NTU) since it combines the advantages of two different illumination modes into one design. The cost of our platform is relatively inexpensive ($45 under low-volume manufacturing, and excluding the smartphone), which can be further improved through large volume manufacturing, benefiting from economies of scale.

**Table 1:** Comparison of the presented turbidimeter with the existing portable turbidimeters.

	Working principle	Hardware					Dynamic range	Time to result	Cost	Ref
		Smartphone based?	Illumination	External photo detector Required?	Sample volume	Battery				
Hussain *et al*.	Proximity sensing	Yes	Infrared LED	No	N/A	Yes	0–400 NTU	N/A	~$86^*^	11
Kelley *et al*.	Light to frequency sensor	No	Infrared LED	Yes	N/A	Yes	0–1000 NTU	N/A	~$100	23
This study	Imaging based	Yes	White LED	No	4 mL	Yes	0.3 NTU – 2000 NTU	- Measurement time: <2 s - Image processing time: <90 s	~$45^*^	—

^*^Excluding the smartphone.

### Future work for potential applications

The presented smartphone-based turbidity reader can be used for a variety of biological and chemical applications. In this work, we demonstrated its use (1) to measure the turbidity of water samples collected from various sources, (2) to measure inoculum density of bacterial samples, (3) to estimate MIC of antibiotics that inhibit the growth of bacteria, and (4) to measure diesel fuel contamination in water samples. This reader can also be used for monitoring the growth of other microorganisms in liquid samples. For example, algae form a potential renewable fuel source, cultivated in wastewater samples, and their growth can be monitored by measuring the turbidity of water samples for high biomass yield^24^. In addition, the toxicity of chemicals (e.g., arsenic, mercury) in wastewater samples might also be determined by monitoring the growth of algae using our reader^25^. Another potential application that may benefit from this portable and cost-effective reader is the estimation of the concentration of viable microorganisms in samples using the most probable number (MPN) method for water and food quality monitoring (e.g., coliform bacteria contamination in water samples and *Enterobacteriaceae* contamination in dairy food products^26^). Among other applications, our turbidity reader can potentially be used as a device to measure the level of calcification propensity in serum samples, which is an important measure of potential risk for cardiovascular disease among patients receiving hemodialysis^27^.

The design of our mobile-phone based reader can be further improved to image multiple cuvettes all at the same time and can be automated to enable time-lapse imaging of samples to minimize operational time and associated costs. Finally, the imaging mode of this reader can be further integrated with other light sources to enable fluorescence based measurements^28^ within the same design.

## Materials and Methods

### Design of the smartphone-based turbidimeter

The portable turbidimeter was developed using a smartphone (Nokia Lumia 1020) and a custom-made opto-mechanical attachment coupled to the rear camera of the smartphone (Fig. 1a). This attachment unit consists of two white LEDs, two coin-size batteries (Product No. B008XBL34A, Amazon), an external lens (Product No. 63–471, Edmund Optics), paper diffusers (Product No. SG 3201, Sphere Optics, Herrsching, Germany), and optical fibers (Product No. ESKA CK-30, Mitsubishi Chemical Corporation, Japan) (Fig. 1b). The LEDs are placed on top of the disposable cuvette (Product No. 60965D-1, Lab Genome, (Houston, TX, USA)). There are four optical fibers (i.e., the transmittance fibers) that are integrated into the unit at the bottom of the sample to capture the transmittance of light and another four optical fibers (i.e. the nephelometric fibers) on the sides of the sample to capture the side-scattered light (Fig. 1c). These fibers are bundled at the other end, facing the external lens of the attachment unit.

The opto-mechanical attachment unit was designed using Autodesk Inventor software and printed using a 3D printer (Stratasys, Dimension Elite). The printed parts were assembled and integrated with the optical components to have the final design (Fig. 1).

### Preparation of the standard curve for turbidity quantification

Since water samples in various environments have different turbidities, a variety of control samples were selected, covering different levels of turbidity. We used standard solutions at 15 NTU, 100 NTU, 750 NTU, and 2000 NTU (Product No. HI88703-11, Hanna Instruments, (Rhode Island, USA)). A sample of NERL Reagent Grade water (Product No. 23-249-580, Fisher Scientific, (Hampton, NH, USA)) was measured to have 0.05 NTU by the Hanna Instruments EPA-compliant benchtop turbidimeter (Product No. HI88703, Hanna Instruments, (Rhode Island, USA)) and used as a reference for the standard solutions. The 2000 NTU sample was diluted to prepare several samples between 100 NTU and 750 NTU (i.e. 180, 240, 280, 320, 370, 400, 450, and 500 NTU) and turbidities were measured with the benchtop gold standard turbidimeter. Finally, lower turbidity samples were also prepared with the dilution of the 100 NTU sample with reagent grade water to test the limits of our turbidimeter. After these samples were prepared, a disposable glass cuvette was filled with 4 mL of each water sample under test and the corresponding vials were imaged.

### Field testing

We collected different water samples (e.g., tap water, pond water, and surface water) from different water sources at UCLA campus. The pond water was collected from a swimming pool at UCLA Sunset Canyon Recreation Center and the surface water samples were collected from UCLA Mildred E. Mathias Botanical Garden.

### Preparation of diesel fuel contaminated water samples

Non-potable cold water was contaminated with diesel fuel (United Oil Gas Station, San Diego, USA) at various concentrations, including 0.005%, 0.01% and 0.05%. First, a solution of 1% diesel fuel was prepared. 100 μL, 200 μL, and 1000 μL of the 1% solution were added to non-potable cold water to a final volume of 20 mL to create diesel fuel contaminated water samples of concentrations 0.005%, 0.01%, and 0.05%, respectively. Non-potable cold water was used as the control. The cuvettes containing each sample were then imaged.

### Preparation of *Escherichia coli (E. coli)* suspensions

We used *E. coli* (ATCC 25922) to prepare bacterial suspensions in phosphate buffered saline (Product No. 20-012-027, Fisher Scientific, Hampton, NH, USA). Plates containing tryptic soy agar plates were inoculated with the bacteria and incubated at 37 °C for 24 h. The bacteria were then collected from the culture agar plates using a disposable loop and suspended at different concentrations in the buffer solution. The concentration of each suspension was measured using a spectrophotometer (ND-ONE-W, ThermoFisher Scientific, Waltham, MA, USA).

### Determination of MIC

We prepared stock solutions of the antibiotics, i.e., ampicillin (Product No. A1593, Sigma-Aldrich, St. Louis, MO, USA) and cephalexin (Product No. PHR1848, Sigma-Aldrich, St. Louis, MO, USA)^19^. 200 mg of ampicillin and 200 mg of cephalexin were each dissolved in 20 mL of reagent grade water to create stock solutions with a concentration of 10,000 mg/L. Next these solutions were diluted to create concentrations of 1000 mg/L, 100 mg/L and 10 mg/L, which were filter sterilized using disposable vacuum filters (Product No. SC50FL025, Fisher Scientific, Hampton, NH, USA).

200 mL of Mueller Hinton Broth (Product No. 70192, Sigma-Aldrich, St. Louis, MO, USA) was prepared and autoclaved for sterilization. For each antibiotic type, 9 vials were labeled based on the dilutions created, namely 32, 16, 8, 4, 2, 1, 0.5, 0.25, 0 mg/L. Antibiotic solution, broth and *E. coli* (ATCC 25922) inoculum were added to each solution. 2000 μL of *E. coli* inoculum with a concentration of 5 × 10^4^ CFU/mL was added to each vial. Broth was then added in the following volumes: 1872 μL to vial 32, 1936 μL to vial 16, 1968 μL to vial 8, 1840 μL to vial 4, 1920 μL to vial 2, 1960 μL to vial 1, 1800 μL to vial 0.5, 1900 μL to vial 0.25 and 2000 μL to vial 0. Subsequently, the corresponding antibiotic solution was added. From the 1000 mg/L stock solution, the following volumes were added: 128 μL to vial 32, 64 μL to vial 16 and 32 μL to vial 8. From the 100 mg/L stock solution, the following volumes were added: 160 μL to vial 4, 80 μL to vial 2 and 40 μL to vial 1. From the 10 mg/L stock solution, the following volumes were added: 200 μL to vial 0.5, 100 μL to vial 0.25, and 0 μL to vial 0. These vials were sealed with a parafilm layer and incubated at 37 °C for 24 h. These resulting vials were then imaged (refer to “Image acquisition” subsection, Materials and Method section for details).

### Image acquisition

After the sample preparation, each disposable cuvette filled with 4 mL of the sample of interest was placed inside our mobile turbidimeter. Two raw format, digital negative (DNG) images per measurement were taken at the image capture settings of daylight, ISO and an exposure time of 0.02 s and 0.8 s (refer to next sub-section for details) using the smartphone based turbidimeter and images were analyzed using a custom developed image processing algorithm. The intensities of the four optical fibers for each mode were then averaged. Three samples were imaged for each data point and the error bars were set to be the positive and negative standard deviation of each turbidity result.

### Optimization of image capture settings

It is important that image overexposure is prevented. Therefore, the optimal exposure times were determined for the transmittance and nephelometry readings. To do this, 0.05 NTU and 2000 NTU samples were prepared. For transmittance readings, the highest light intensity appeared for the lowest turbidity level, 0.05 NTU. Therefore, this sample was used to determine an appropriate exposure time to eliminate overexposure. For nephelometric readings, the 2000 NTU sample was used because it scatters strongly.

These two samples, 0.05 NTU and 2000 NTU, were imaged at all exposure times available on the camera from 1/200 s to 4 s. The raw DNG image files were converted to 8-bit Tagged Image File Format (TIFF). Then, using custom image processing algorithms, all the channels including R, G, B, H, S, V, Y, Cb, and Cr were extracted and the maximum intensity in the image was determined for each channel. If the light intensity was 255, the image was overexposed, and a lower exposure time had to be used. Therefore, the optimal exposure time for each channel was determined by taking the highest exposure time that had a maximum intensity under 255.

It is also important that an appropriate color channel is selected to maximize the differences seen between the low turbidity and high turbidity samples. This was determined using a custom algorithm that calculated and maximized the difference in the average intensity between the 0.05 NTU sample and 2000 NTU sample. This algorithm first converted images to 8-bit TIFF files. Then, all the image channels were extracted from the appropriate image, depending on the optimal exposure time for that channel and mode of measurement (transmission or nephelometric). If the image was intended to analyze light transmission, then the four transmission optical fibers were cropped. Otherwise if the image was intended to analyze light scattering, then the four nephelometric optical fibers were accordingly cropped. Intensities of these four optical fibers were averaged. The differences in the average intensities of 0.05 NTU and 2000 NTU samples were calculated and the color channel that provided the maximum difference was selected as the optimum color channel. Based on this analysis, the blue channel of the image captured with 0.02 s exposure time and the green channel of the image captured with 0.8 s exposure time were selected to be optimum for transmittance and nephelometry, respectively.

### Image analysis

We used a custom developed algorithm that handles all the image processing steps automatically. It detects the transmittance and nephelometry fibers in an image and gives out two intensity values (i.e. I_T_ and I_R_) that can be used in Eq. (1) to determine the turbidity of the sample under test.

Raw format images were first converted to 8-bit TIFF. The blue channel of the image with 0.02 s exposure time was extracted for the transmittance mode and the green channel of the image with 0.8 s exposure time was extracted for the nephelometric mode (refer to the previous sub-section for details). To find the exact location of each fiber in a given image, the intensity of the image was multiplied by 5 and the image was resized to decrease the computation time. The circular area of each fiber end was found by searching circles with a diameter of 10–40 pixels in the resized image. If there were any duplicate circles detected within a diameter of 20 pixels or less, then the average coordinates of these circles were calculated to find the exact coordinates of the corresponding fiber center. Then, the images of four optical fibers were cropped out using a mask for each mode of operation and their intensities were averaged (Fig. 2). The identified intensity values (I_T_ and I_R_) are used in Eq. (1) to quantify the turbidity of the sample.

### Operation of the device

After the sample collection from the water source, the disposable glass cuvette containing 4 mL of the sample is put into the holder of the portable turbidimeter and the cuvette is covered with a lid containing the LEDs (Fig. 1b). The white LEDs are turned on and the images of fiber-ends are captured sequentially using the smartphone. The captured images are then sent to our servers using a custom-developed smart application and processed using our image processing algorithm to quantify the turbidity of the sample. The turbidity value of the sample (in NTU) is sent back to the display of the smartphone through the same application.

### Smartphone application

We created a smartphone application to assist the users in taking new measurements and reviewing past measurements on the phone. The application allows the users to select two raw format images from the library and transmit them to our server. The server then performs image processing using the custom developed image processing algorithm, detailed earlier. It converts the raw format images to TIFF, calculates the mean intensity values of the transmission and nephelometry fibers. The calculated turbidity value in NTU (based on Eq. (1)) is then transmitted back to the smartphone, displayed through the same custom-developed application. The user may tap the “View History” menu option in the application to list all the past samples and their results, with the most recent jobs listed first. A copy of the images and the corresponding turbidity results are also maintained on the servers.

## Supplementary information

Supplementary Information
