# Supplementary material for: Smartphone-based turbidity reader
Source: Sci Rep. 2019 Dec 27;9:19901. doi: 10.1038/s41598-019-56474-z (PMC6934863; doi:10.1038/s41598-019-56474-z)
Supplement: Supplementary file 1 — Supplementary Information [file 41598_2019_56474_MOESM1_ESM.pdf]

Supplementary Information

Smartphone-based turbidity reader

Hatice Ceylan Koydemir[1,2,3,†], Simran Rajpal[1,†], Esin Gumustekin[4], Doruk Karinca[5], Kyle Liang[5],

Zoltan Gorocs[1,2,3], Derek Tseng[1,2,3], and Aydogan Ozcan[1,2,3,*]

[1]Biengineering Department, University of California Los Angeles, 90095, CA, USA

[2]Electrical and Computer Engineering Department, University of California, Los Angeles, 90095, CA, USA

[3]California NanoSystems Institute, Los Angeles, 90095, CA, USA

[4]Microbiology, Immunology and Molecular Genetics, University of California Los Angeles, 90095, CA, USA

[5]Computer Science and Engineering Department, University of California, Los Angeles, 90095, CA, USA

†These authors contributed equally to this work.

*Corresponding author: ozcan@ucla.edu

**Figure S1**	Schematics illustrating the mechanism of (a) nephelometric turbidity measurement, (b) transmittance based turbidity measurement for a liquid sample in a disposable glass cuvette.
**Table S1**	Calibration data used in Figure 4.
**Table S2**	Precision and accuracy data of the reader at different turbidity levels.

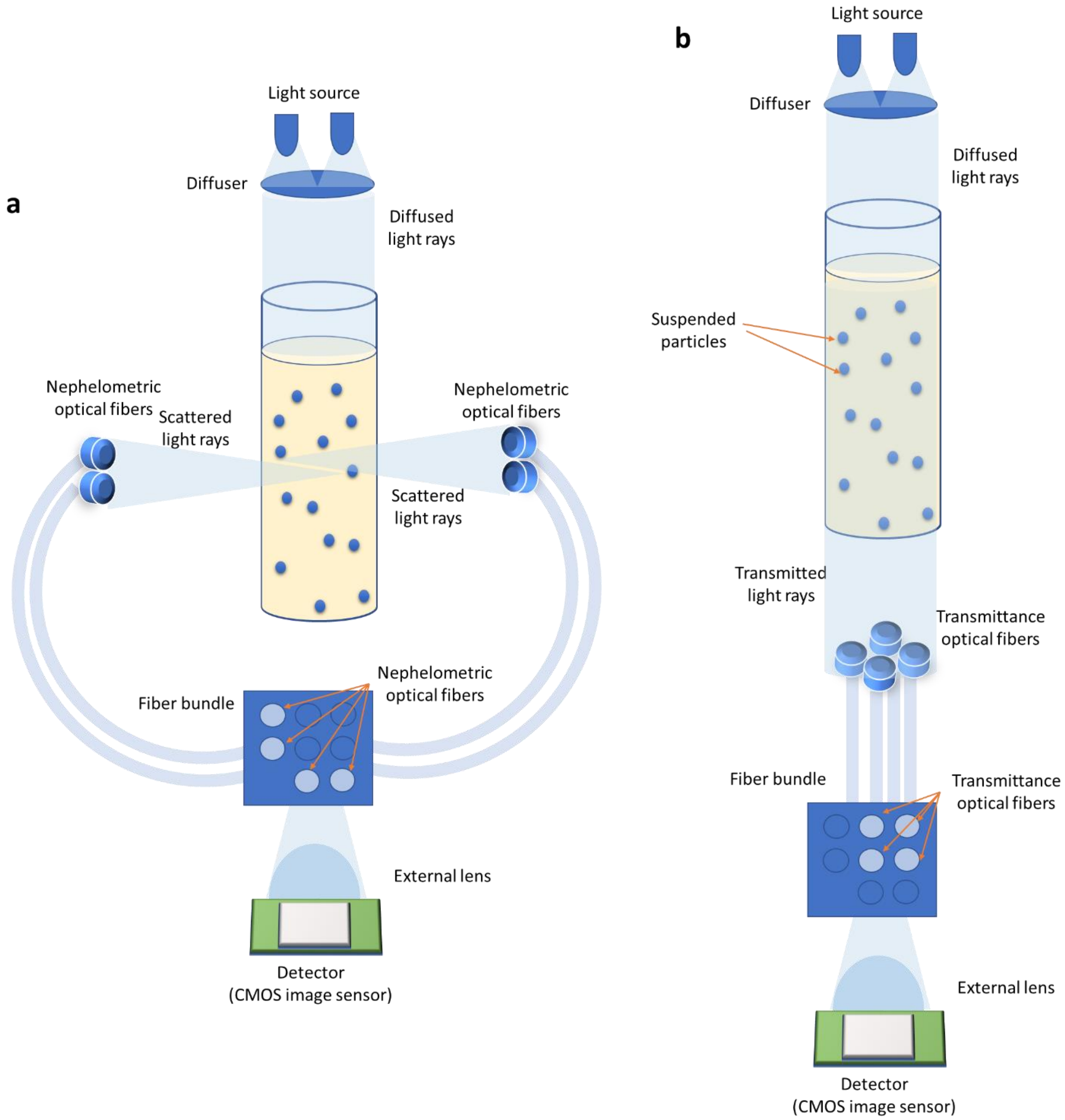

Figure S1. Schematics illustrating the mechanism of (a) nephelometric turbidity measurement, (b) transmittance based turbidity measurement for a liquid sample in a disposable glass cuvette.

Table S1. Calibration data used in Figure 4.

NTU	I_R	I_T	$I_{R,\sigma}$	$I_{T,\sigma}$
0.05	6.4413	133.5465	0.1993	1.0363
0.8	8.5412	170.2480	0.0838	3.5043
0.95	10.0402	159.0785	0.3041	3.9844
1.4	10.5031	158.3732	0.1127	6.5292
2.1	11.3889	162.3609	0.2578	4.2214
3.3	12.2540	164.0013	0.2094	3.2994
7.1	14.3217	173.1743	0.4304	3.7294
8	13.3665	146.1655	0.2927	2.4395
10	16.1618	163.9319	0.4321	4.0656
12	15.79586	144.218	0.2319	6.3517
15	16.41719	142.3204	0.1409	1.3350
100	51.53643	124.3573	0.5966	1.4549
180	75.0622	131.2323	1.0467	5.6458
240	91.85185	106.7126	1.0392	6.6322
280	102.3085	90.90978	1.9113	5.2107
320	107.5798	71.45283	0.7959	5.4751
370	132.3294	63.38244	0.7793	3.0877
400	142.2062	53.45454	0.5336	2.2884
450	150.6569	41.62234	0.8552	3.3755
500	162.7766	33.93486	0.4345	1.5979
750	162.0428	11.1522	0.7980	0.4009
2000	121.401	12.35747	4.1239	0.8230

σ: standard deviation
I: average intensity

Table S2. Precision and accuracy data of the reader at different turbidity levels.

True Value (NTU)	Calculated Mean Turbidity (NTU)	Standard Deviation
0.05	0.24	0.02
0.8	0.60	0.02
0.95	1.14	0.14
1.4	1.37	0.07
2.1	2.01	0.22
3.3	4.01	0.44
7.1	8.38	0.91
8	6.36	0.62
10	12.26	0.91
12	11.49	0.49
15	12.80	0.30
100	114.32	1.89
180	188.80	3.31
240	241.96	3.29
280	275.07	6.05
320	294.49	21.96
370	331.52	16.47
400	393.94	17.16
450	510.39	41.48
500	625.32	30.01
2000	1748.86	114.23
